# Spectroscopy of Filled Single-Walled Carbon Nanotubes

**DOI:** 10.3390/nano12010042

**Published:** 2021-12-23

**Authors:** Marianna V. Kharlamova, Christian Kramberger

**Affiliations:** 1 Institute of Materials Chemistry, Vienna University of Technology, Getreidemarkt 9/BC/2, 1060 Vienna, Austria; 2 Moscow Institute of Physics and Technology, Institutskii Pereulok 9, 141700 Dolgoprudny, Russia; 3 Centre for Advanced Material Application (CEMEA) of Slovak Academy of Sciences, Dúbravská cesta 5807/9, 854 11 Bratislava, Slovakia; 4 Faculty of Physics, University of Vienna, Strudlhofgasse 4, 1090 Vienna, Austria

**Keywords:** single-walled carbon nanotube, filling, spectroscopy, optical absorption spectroscopy, Raman spectroscopy, photoemission spectroscopy, X-ray absorption spectroscopy

## Abstract

Many envisaged applications, such as nanoelectronics, photovoltaics, thermoelectric power generation, light-emission devices, energy storage and biomedicine, necessitate single-walled carbon nanotube (SWCNT) samples with specific uniform electronic properties. The precise investigation of the electronic properties of filled SWCNTs on a qualitative and quantitative level is conducted by optical absorption spectroscopy, Raman spectroscopy, photoemission spectroscopy and X-ray absorption spectroscopy. This review is dedicated to the description of the spectroscopic methods for the analysis of the electronic properties of filled SWCNTs. The basic principle and main features of SWCNTs as well as signatures of doping-induced modifications of the spectra of filled SWCNTs are discussed.

## 1. Introduction

Single-walled carbon nanotubes (SWCNTs) can be either metals or semiconductors, solely dependent on their atomic structure, also known as chirality. Many envisaged applications, such as nanoelectronics [1], photovoltaics [2], thermoelectric power generation [3], light-emission [4], energy storage [5] and biomedicine [6], necessitate nanotube samples with specific uniform electronic properties. The methods of laboratory-scale chirality selective synthesis and separation of SWCNTs [7] were recently developed. There are also alternative more scalable approaches of post-synthetic chemical functionalization of SWCNTs, which allow for the controlled modification of the electronic properties of SWCNTs [8,9]. One example is the endohedral functionalization (filling) of SWCNTs. It represents a viable and flexible approach to fine-tune their electronic properties, because a large variety of substances with appropriate physical and chemical properties can be introduced inside SWCNTs [10]. The encapsulation of electron donor or acceptor substances inside SWCNTs opens the way of Fermi level engineering of SWCNTs for specific applications.

The precise investigation of the electronic properties of filled SWCNTs on a qualitative and quantitative level is conducted by the state-of-the-art spectroscopic techniques. Among them are optical absorption spectroscopy (OAS), Raman spectroscopy (RS), photoemission spectroscopy (PES) and X-ray absorption spectroscopy (XAS). 

This review is dedicated to the description of the spectroscopic methods for the analysis of the electronic properties of filled SWCNTs. The review includes the discussion of basic principle and main features of the spectra of SWCNTs as well as signatures of doping-induced modifications of the spectra of filled SWCNTs. In Section 2.1 the results of the optical absorption investigations of filled SWCNTs are considered. Section 2.2 discusses the results of the Raman spectroscopy investigations of filled SWCNTs. In Section 2.3 the results of the photoemission studies of filled SWCNTs are presented. Section 2.4 is dedicated to the description of the X-ray absorption spectroscopy data of filled SWCNTs. 

## 2. Methods of the Investigation of the Electronic Properties of Filled SWCNTs

### 2.1. Optical Absorption Spectroscopy

#### 2.1.1. Basic Principle and Main Features of the OAS Spectra of SWCNTs

Optical absorption spectroscopy investigates the absorption of light by matter. The method is based on the illumination of a transparent sample (in the form of solid or solution) with monochromatic light and measuring the spectrum of the transmitted light. The incoming light can have wavelengths in the near ultraviolet (200–380 nm), visible (380–780 nm) and near-infrared (780–3000 nm) spectral ranges. The absorption of light with these wavelengths is due to electronic transitions from the electronic ground state to an excited state in substances. The transitions between electronic energy levels define the positions of absorption bands in the optical absorption spectra. Additionally, vibrational and rotational energy levels contribute to the shapes of the absorption bands forming their fine structure [11]. 

When a light beam passes through a transparent cuvette with a sample solution, a part of the beam is absorbed and the other part is transmitted. According to Lambert’s law, the relative quantity of the absorbed and transmitted light does not depend on the intensity of the incoming light: each layer of a sample solution of equal thickness absorbs an equal fraction of the incoming light [11]. The Lambert’s law can be thus expressed in the following form:

*I* = *I*_0_*e*^−*βl*^ (1)

where *I*_0_ is the intensity of the incoming light, *I* is the intensity of the transmitted light, *β* is the absorption coefficient, *l* is the optical path through the solution [11]. The dependence *I*(*l*) is represented graphically as an exponential curve. 

The transmittance of the sample solution (the relative amount of the transmitted light) is written as:

*T*_s_ = *I*/*I*_0_ = *e*^−*βl*^ (2)

Therefore, the absorptance of the sample solution (the relative amount of the absorbed light) is given by the equation:

*a*_s_ = (*I*_0_ − *I*)/*I*_0_ = 1 − *T*_s_ = 1 − *e^−βl^* (3)

The transmittance and absorptance are usually expressed in % and range from 0 to 100% [11]. 

In many cases, it is convenient to use power of 10 instead of power of *e* in Equation (1). Then the transmittance of the sample solution is presented in the form:

*T*_s_ = 10^−*As*^ (4)

where *A_s_* is the absorbance or optical density. It is connected with the intensities of the incoming and transmitted light by the following equation:

*A_s_* = −log (*I*/*I*_0_) (5)

Taking into consideration Equations (2) and (4), we obtain [11]:

*A_s_* = −log *T*_s_ (6)

The absorbance is usually expressed as a decimal fraction. One hundred percent transmission of light through the sample corresponds to zero absorption. The optical absorption spectrum represents the dependence of the absorbance on the wavelength of the incoming light.

According to the Beer–Lambert law, the absorbance of the solution is directly proportional to the concentration of a substance in the solution:

*A*_s_ = *εcl* (7)

where *ε* is the molar absorption coefficient (or the extinction coefficient), *c* is the molar concentration of the solution [11]. Graphically, the dependence *A*(*c*) is linear and goes through the origin of coordinates (when the optical path through the solution *l* is constant). The linear dependence is obtained only for solutions where physical and chemical properties do not depend on the concentration. 

**Figure 1:**
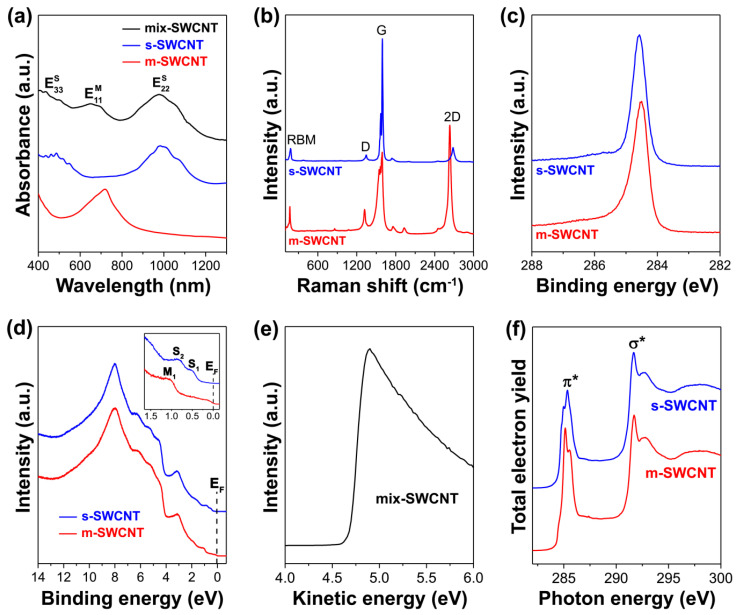
(**a**) The OAS spectra of 1.4 nm-diameter metallicity-mixed (mix-SWCNT) (Reprinted by permission from [13]: SpringerNature, JETP Letters, Kharlamova et al. Study of the electronic structure of single-walled carbon nanotubes filled with cobalt bromide, copyright 2010), semiconducting (s-SWCNT) [25] and metallic SWCNTs (m-SWCNT) [25]. $E_{22}^{S}$, $E_{33}^{S}$ and $E_{11}^{M}$ denote the peaks that correspond to electronic transitions between the second, the third vHs of semiconducting SWCNTs and the first vHs of metallic SWCNTs. (**b**) The Raman spectra of 1.4 nm-diameter semiconducting [25] and metallic SWCNTs [25] acquired at laser energies of 2.41 eV (λ_ex_ = 514 nm) and 1.96 eV (λ_ex_ = 633 nm), respectively. The radial breathing mode (RBM), D, G and 2D-bands of Raman spectra are indicated. (**c**) The C 1s XPS spectra of 1.4 nm-diameter semiconducting [25] and metallic SWCNTs [25]. (**d**) The UPS spectra of 1.4 nm-diameter semiconducting [25] and metallic SWCNTs [25]. The inset zooms in the peaks that originate from the first and the second vHs of semiconducting SWCNTs (S_1_ and S_2_, respectively) and the first vHs of metallic SWCNTs (M_1_). E_F_ denotes the Fermi level of SWCNTs. (**e**) The secondary electrons cutoff spectrum of metallicity-mixed SWCNTs [14]. (**f**) The XAS spectra of 1.4 nm-diameter semiconducting [26] and metallic SWCNTs [26]. Copyright 2009, American Physical Society. The π*- and σ*- resonances are indicated.

The optical absorption spectroscopy was applied to identify the presence of charge transfer in the SWCNTs filled with FeCl_2_, FeBr_2_, FeI_2_ [12], CoBr_2_ [13], ZnCl_2_, ZnBr_2_, ZnI_2_ [14], AgCl, AgBr, AgI [15], CdCl_2_, CdBr_2_, CdI_2_ [16], CuCl [17], CuCl, CuBr, CuI [18], PrCl_3_ [19], TbCl_3_ [20], GaSe, GaTe [21,22], SnS, SnTe [22,23], Bi_2_Se_3_ [22] and Bi_2_Te_3_ [24]. The optical absorption spectrum of SWCNTs includes several characteristic peaks. These peaks originate from electronic transitions between van Hove singularities (vHs) in the valence and conduction bands of semiconducting and metallic SWCNTs. Figure 1a shows the OAS spectra of 1.4 nm-diameter metallicity-mixed and sorted SWCNTs [13,25]. Three peaks are observed in the spectrum of metallicity-mixed SWCNTs. The first peak at 1.20 eV (1030 nm) corresponds to the $E_{22}^{S}$ electronic transitions between the second vHs of semiconducting SWCNTs. The second peak at 1.72 eV (710 nm) belongs to the $E_{11}^{M}$ electronic transitions between the first vHs of metallic SWCNTs. The third peak at 2.89 eV (430 nm) originates from the $E_{33}^{S}$ electronic transitions between the third vHs of semiconducting SWCNTs [13]. The spectrum of semiconducting SWCNTs shows the $E_{22}^{S}$ and $E_{33}^{S}$ optical absorption bands [25]. No $E_{11}^{M}$ optical absorption bands due to metallic transitions are observed. The spectrum of metallic nanotubes shows only the M_11_ band, and no bands of semiconducting SWCNTs are visible [25].

#### 2.1.2. Signatures of Doping-Induced Modifications of the OAS Spectra of SWCNTs

**Figure 2:**
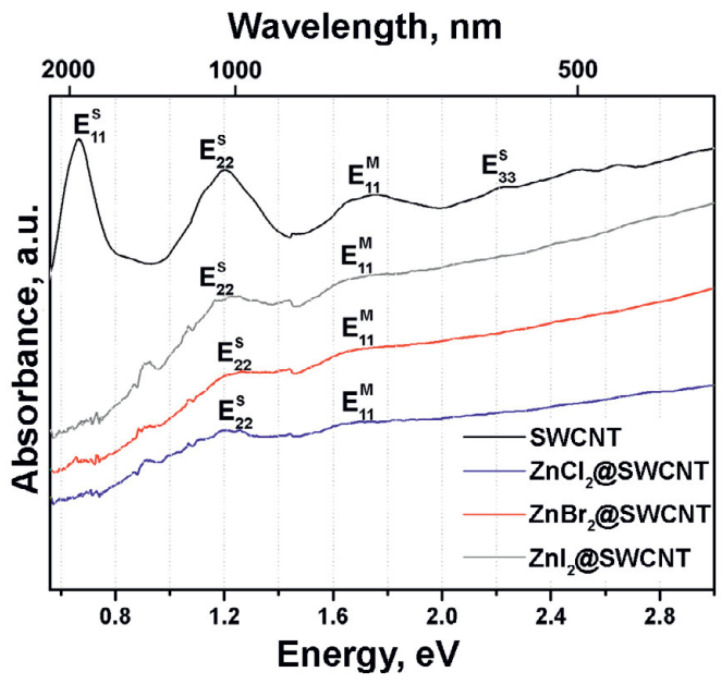
The OAS spectra of the pristine SWCNTs and nanotubes filled with zinc halogenides. Reprinted by permission from [14]: SpringerNature, European Physical Journal B, Kharlamova et al. Acceptor doping of single-walled carbon nanotubes by encapsulation of zinc halogenides, copyright 2012.

Upon filling-induced doping of SWCNTs, their OAS spectrum can undergo suppression of the characteristic peaks (Table 1). This is due to the depletion of optical transitions between the vHs in the valence band and conduction band of SWCNTs, because of emptying the vHs in the valence band or occupying the vHs in the conduction band (Figure 2) [14]. This effect is caused by the charge transfer-induced down- or upshift of the Fermi level of SWCNTs, respectively, i.e., *p*-type or *n*-type doping of SWCNTs [10]. However, the type of doping could not be identified from the OAS data.

#### 2.1.3. Further Developments of the OAS Spectroscopy of Carbon Nanotubes

The optical properties of carbon nanotubes are important for developing opto-electronic components of devices. The optical absorption investigations of carbon nanotubes and filled nanotubes are needed to know the optical properties for applications. The increase of accuracy, spatial resolution, intensity of signal as well as performing the quantitative analysis of spectra would be the next steps.

### 2.2. Raman Spectroscopy

#### 2.2.1. Basic Principle and Main Features of the Raman Spectra of SWCNTs

Raman scattering is the inelastic scattering of light with an energy transfer between the photon and a quasi-particle representing an oscillating inhomogeneity. Depending on the type of matter, quasi-particles can be optical phonons, plasmons, optical magnons and electronic excitations (in solids) [27] or molecular vibrations (in gases, liquids and molecular solids) [28].

Raman spectroscopy is the technique based on the illumination of a sample with monochromatic light and measuring the spectrum of the inelastically scattered light. The Raman shift that is detected corresponds to a change in the frequency of scattered photons as compared to incident photons and is typically expressed in wavenumbers (cm^−1^).

The inelastic scattering of monochromatic radiation was predicted theoretically by the Austrian physicist A. Smekal in 1923 [29]. The first experimental observations of the Raman scattering were reported by the Indian scientists C.V. Raman and K.S. Krishnan [30] and the Soviet scientists G. Landsberg and L. Mandelstam [31] in 1928. However, the low intensity of the inelastic scattering as compared to Rayleigh scattering impeded the development of Raman spectroscopy. The invention of the laser in 1960 [32], which was soon employed as a monochromatic source for the method [33], removed these restrictions. Further developments of laser sources, monochromators, detectors and optics for Raman spectrometers, as well as techniques for the data processing enabled obtaining high quality data and made Raman spectroscopy a powerful method for the characterization of many different materials [28].

**Figure 3:**
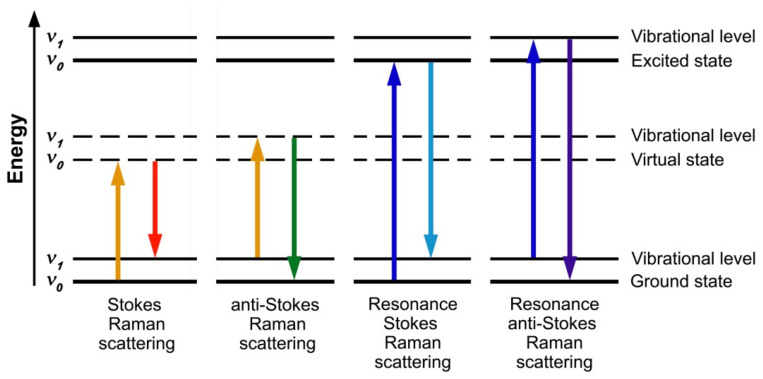
The schematics of Raman scattering processes of photons. The arrows show the transitions between different states, which are labeled; *n* and *m* denote the vibrational ground and excited states.

The Raman scattering process can be understood by considering the absorption of light by a molecule. The light absorption leads to a transition of the molecule from its ground state to an excited state. In this case, the energy of the incident photon corresponds to the difference between energies of the ground and the excited states [28,34]. When the molecule relaxes from the excited to the ground state, a photon with the same energy is emitted. In Raman scattering, there is an energy exchange between the photon and the molecule that leads to a change in the rotational and vibrational state of the molecule. Figure 3 shows the schematics of Raman scattering processes. The photon interacts with the molecule and polarizes its electron cloud around the atomic nuclei with the formation of a short-lived “virtual” state. This state is unstable and the photon with a changed energy is re-radiated [34]. The scattered photon may have lower or higher energy than the incident photon. In the first case, which is called Stokes scattering, the molecule is transited from the ground vibrational state *n* to a “virtual” state with the absorption of energy and is promoted to an excited vibrational state *m* with a higher energy. In the second case, which is called anti-Stokes scattering, the molecule that is already in an excited state such as *m* due to the thermal fluctuations is transited to the ground state *n* (Figure 3) [28,34]. Because the number of molecules that are in an excited vibrational state at room temperature is small, anti-Stokes scattering is weak as compared to Stokes scattering [34]. When the energy of the incident photon is close to the energy of an electronic transition between the ground and the excited states of the molecule, resonance Raman scattering occurs (Figure 3). In this case, the Raman scattering intensities can be enhanced by several orders of magnitude [27].

Raman spectroscopy was applied to investigate the modified electronic structure of SWCNTs filled with MnCl_2_, MnBr_2_ [35,36], FeCl_2_, FeBr_2_, FeI_2_ [12], CoBr_2_ [13], NiCl_2_, NiBr_2_ [37], ZnCl_2_ [20], ZnCl_2_, ZnBr_2_, ZnI_2_ [14], AgCl, AgBr, AgI [15], AgCl [38,39], CuCl [17], CuI [40,41], CuCl, CuBr, CuI [18], CdCl_2_ [20,42], CdCl_2_, CdBr_2_, CdI_2_ [16], PbCl_2_, PbBr_2_, PbI_2_ [43], SnF_2_ [44], RbI [45], RbAg_4_I_5_ [46], TbCl_3_ [20,47,48], TbBr_3_, TbI_3_ [48], TmCl_3_ [24,47], PrCl_3_ [19,47], LuCl_3_, LuBr_3_, LuI_3_ [49], HgCl_2_ [50], GaSe, GaTe [21,22], SnS, SnTe [22,23], Bi_2_Se_3_ [22] and Bi_2_Te_3_ [24], Ag [24,51,52,53], Cu [53,54], ferrocene [55,56,57,58], cobaltocene [59,60] and nickelocene [61,62,63].

The Raman spectrum of SWCNTs contains four main regions: the radial breathing mode (RBM) at frequencies below 300 cm^−1^, which is assigned to radial vibrations of carbon atoms, the D-band at 1300–1400 cm^−1^, which is enabled at structural defects and disordering, the G-band at 1500–1700 cm^−1^, which belongs to the longitudinal and transversal phonon and the 2D-band at 2500–2800 cm^−1^, which is the overtone of the D-line [64]. The RBM-band includes several peaks, whose positions ($\omega_{RBM}$) are inversely proportional to the SWCNT diameter by the equation:

(8) $$\omega_{RBM}=\frac{227}{d_{t}}\sqrt{1+Cd_{t}^{2}},$$

where *C* = 0.05786 nm^−2^ [65], and thus characterize the diameter distribution of nanotubes. The G-band includes three components G^–^_LO_, G^+^_TO_ and G^+^_LO_. The G^–^_LO_-component at 1550 cm^−1^ corresponds to longitudinal optical (LO) phonon in metallic SWCNTs. The G^+^_TO_-component at 1570 cm^−1^ and G^+^_LO_-component at 1590 cm^−1^ belong to transversal (TO) and longitudinal optical phonon in semiconducting SWCNTs, respectively [66]. The profiles of the G-band of Raman spectra of semiconducting and metallic SWCNTs are different. The G-band of semiconducting SWCNTs has a narrow Lorentzian shape, whereas the G-band of metallic nanotubes has a broad asymmetric Breit–Wigner–Fano shape, because of the intense G^–^_LO_-component [64,67]. Figure 1b demonstrates the Raman spectra of 1.4 nm-diameter semiconducting SWCNTs (acquired at a laser wavelength of 514 nm) and metallic SWCNTs (acquired at a laser wavelength of 633 nm) [25].

#### 2.2.2. Signatures of Doping-Induced Modifications of the Raman Spectra of SWCNTs

**Figure 4:**
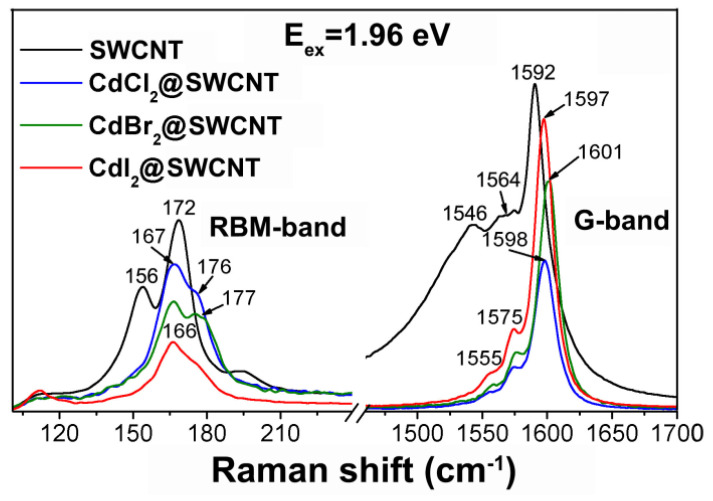
The RBM and G-bands of Raman spectra of the pristine SWCNTS and nanotubes filled with cadmium chloride, cadmium bromide and cadmium iodide acquired at laser energy 1.96 eV. Reprinted by permission from [16]: SpringerNature, Journal of Materials Science, Kharlamova et al. Charge transfer in single-walled carbon nanotubes filled with cadmium halogenides, copyright 2013.

Upon filling-induced doping of SWCNTs, their Raman spectra undergo such alterations as shifts and changes of relative intensities of the peaks of the RBM and G-bands (Figure 4) [16]. These alterations depend on the type of doping and differ for metallic and semiconducting SWCNTs. Table 2 summarizes the doping-induced modifications of the Raman spectra of filled SWCNTs.

In the literature, there are several mechanisms proposed for explaining doping-induced changes of the G-band of Raman spectra of SWCNTs. For instance, the shifts in the G-band upon electrochemical doping were reported to be caused by a combination of (i) nonadiabatic (dynamic) effects due to electron–phonon coupling and (ii) (static) lattice relaxation effects [68]. Populating the conduction band or depleting the valence band affects the screening in the electronic system and reduces the renormalization in the coupled phonons. In either case, *p*- or *n*-doping, the observed effect is an upshift of the G_TO_ and G_LO_-modes. Another effect is the change of the C-C bond length and strength with adding or removing electrons. The bonds will contract upon *p*-doping and this will in turn result in an additional upshift of the G_TO_ and G_LO_-modes [69]. The additive behavior of the two effects will always lead to an upshift of the G-band of semiconducting SWCNTs [68,70,71,72,73]. Due to the stronger electron–phonon coupling in the TO phonon in semiconducting SWCNTs, the G_TO_-mode shows always a larger upshift than the G_LO_-mode [68,70,73]. In case of *n*-doping, the renormalization is opposed by C−C bond softening. The resulting upshift is diminished as compared to *p*-doping. At very high doping levels the bond softening can even overcompensate the renormalization and lead to an effective downshift in the G-line [72]. The two opposing effects depend differently on the diameter of the SWCNTs. On the one hand, electron–phonon coupling is stronger at larger diameters and smaller curvatures [70]. On the other hand, C−C bond weakening is stronger at smaller diameters [72]. Therefore, in semiconducting SWCNTs *n*-doping may result in a diminished upshift or even a downshift of the G-band depending on the doping level and the diameter.

The G^–^_LO_-mode (Breit–Wigner–Fano mode) in the G-line of metallic SWCNTs is also affected by doping, but the underlying mechanism differs greatly. In the pristine metallic SWCNTs, there is a Kohn anomaly in the phonon dispersion, which results in a sizable phonon softening of the LO mode [74,75]. The Kohn anomaly is due to the enhanced electron–phonon coupling of the LO phonon with electrons near the Fermi point [74,75]. Doping-induced changes in the Fermi level (E_F_) strongly affect the electron–phonon coupling and have also an immediate effect on the downshift and also the linewidth of the metallic LO mode [76]. Either *p*- or *n*-doping weakens the electron–phonon coupling. In both cases, the observed G^–^_LO_-peak shifts upwards and the Fano parameter in the characteristic Breit–Wigner–Fano lineshape is reduced. As the G^–^_LO_-peak is upshifted, it also becomes more symmetric and narrow [70,71,73,77,78].

The RBM profile in the Raman spectra of SWCNTs crucially depends on the resonance conditions. It is therefore commonly, without an underlying mechanism, proposed that doping can affect the resonance conditions for individual chiralities resulting in the origin of the shifts, alterations of relative intensities, disappearance or appearance of RBM peaks [14,16,47].

#### 2.2.3. Further Developments of Raman Spectroscopy of Carbon Nanotubes

Raman spectroscopy gives information about the electronic properties of carbon nanotubes that is necessary for applications. The processing of Raman spectra allows deducing the quantitative information. The further development of advanced Raman spectroscopy techniques such as surface- and tip-enhanced Raman spectroscopy and Raman imaging is required to increase the accuracy and resolution for collecting the versatile information about the electronic properties of filled carbon nanotubes.

### 2.3. Photoemission Spectroscopy

#### 2.3.1. Basic Principle and Main Features of the PES Spectra of SWCNTs

Photoemission spectroscopy is based on the irradiation of the surface of a sample with photons and analyzing of the energies of the ejected photoelectrons. In X-ray photoelectron spectroscopy (XPS), X-ray radiation is used, and the kinetic energy and number of photoelectrons ejected from core levels of atoms are measured [79]. The X-ray photons emitted by an X-ray gun have the energies of greater than 1 keV. The Al K_α_ X-ray radiation with photon energy of 1486.6 eV and Mg K_α_ X-ray radiation with photon energy of 1253.6 eV are typically used. In ultraviolet photoelectron spectroscopy (UPS), ultraviolet radiation with the energies of photons of tens of eV is used, and the kinetic energy and number of photoelectrons ejected from shallow valence band levels of atoms are analyzed [80]. In the laboratory spectrometers, ultraviolet photons are produced using a gas discharge lamp, typically filled with helium. The photons emitted by helium gas have energies of 21.2 eV (He I) and 40.8 eV (He II).

**Figure 5:**
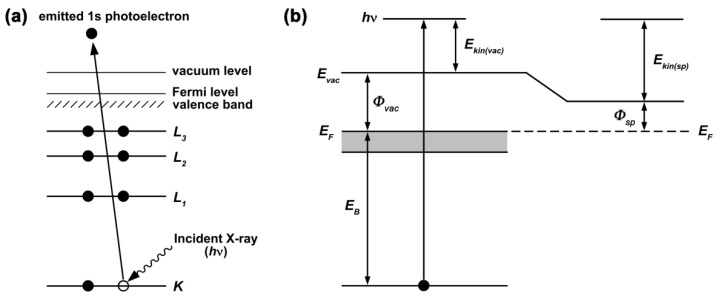
(**a**) The schematics of the photoemission process where a 1s photoelectron is ejected from the K core level of the atom upon irradiation with X-ray photons. The energy levels are labeled. (**b**) The energy level diagram for the photoemission process into vacuum and into the spectrometer. *E_F_* is the Fermi level, the highest filled states below *E_F_* are colored in grey, *E_vac_* is the vacuum level, *hν* is the energy of the incident X-ray photon that is transferred to the photoelectron, *E_B_* is the binding energy of the photoelectron, *Φ_vac_* and *Φ_sp_* are the work functions relative to the vacuum level and the spectrometer level, respectively, *E_kin(vac)_* and *E_kin(sp)_* are the kinetic energies of the photoelectron in vacuum and measured by the spectrometer, respectively.

The photoemission process is illustrated in Figure 5a, where a 1s photoelectron is ejected from the K shell of the atom. XPS and UPS are the methods for the surface analysis of samples, because a mean free path of emitted photoelectrons in solids equals several nanometers. Therefore, the thickness of the surface layer investigated in XPS is up to 10 nm. The lower incident photon energies used in UPS lead to the emission of photoelectrons of much lower kinetic energies than those measured in XPS, therefore giving an approximate information depth of 2–3 nm.

The XPS and UPS measurements are performed in an ultra high vacuum (better than 10^−9^ mbar), because an analytical signal from low-energy photoelectrons can be scattered on residual gas molecules and they can be quickly adsorbed on the surface of the sample [79].

The XPS and UPS spectra represent the dependence of number of detected electrons (expressed as counts or counts/s) on their energy. The latter can be expressed in the kinetic energy scale (*E_k_*), which is measured during the experiment, but depends on the photon energy of the radiation and is thus not an intrinsic property of an investigated material. The energy can also be expressed in the binding energy scale (*E_B_*), which is characteristic of atomic levels of chemical elements [79].

The photoelectric effect was discovered by H.R. Hertz in 1887 [81]. He observed the emission of electrons from the surface of metal under irradiation with ultraviolet light. The theoretical explanation of the photoelectric effect was provided by A. Einstein in 1905 [82]. He obtained the fundamental photoelectric equation that is the relation between the energy of incoming photon *hν* and the maximum kinetic energy of the emitted photoelectrons $E_{kin}^{\max}$:

(9) $$E_{kin}^{\max}=h\nu -\Phi_{0}$$

where *Φ*_0_ is a characteristic constant of the surface of the sample and is called today the work function. In 1907, P.D. Innes observed the emission of photoelectrons from metals using a Röntgen tube fitted with a platinum anode that generated X-rays [83]. Later on, H. Moseley, W. Rawlinson and H. Robinson performed a set of XPS experiments, and by the early 1920s, the photoelectron spectra of different elements were obtained. These works were conducted using a variety of high-energy X-rays, magnetic analysis of photoelectrons and their photographic detection [84]. In 1953, R.G. Steinhardt firstly considered XPS as a potential analytical tool for investigating surface chemical processes such as corrosion and catalysis [85]. The breakthrough in XPS was made by K. Siegbahn in 1957. His research group performed modernizations of the XPS equipment that led to an improved resolution of the spectra and allowed for accurate determination of core level binding energies [86]. In 1967, K. Siegbahn published the book where all aspects of the XPS technique were covered, including the physical principles, instrumental design and extensive collected data [87]. The book demonstrated a large potential of XPS and attracted an interest of instrument manufacturers. In 1969, the first commercial XPS spectrometers were produced. Further developments of X-ray sources, monochromators, analyzers and detector systems allowed for obtaining high quality data and conducting different types of analysis for certain purposes [88].

The UPS technique was pioneered by F.I. Vilesov in 1961 to study the photoelectron spectra of free molecules in the gas phase [89]. In these experiments, monochromatized ultraviolet radiation from a hydrogen discharge and a retarding potential analyzer were used. The method was further developed by several groups, in particular by the group of W. Spicer, who measured the first valence band spectrum of metals [90,91], and the group of D. Turner, who conducted UPS on gases [92] using a new type of ultraviolet radiation source, namely the differentially pumped gas discharge lamp [93] that is still employed in UPS spectrometers today.

The kinetic energy of the photoelectron measured by the spectrometer *E_kin_*_(*sp*)_ equals the energy of incident photon *hν* reduced by the sum of the binding energy of the photoelectron *E_B_* relative to the Fermi level (*E_F_*) of the sample (defined as *E_F_* = 0) and the work function of the solid relative to the spectrometer level *Φ_sp_*. It can thus be written in the following form:

*E*_*kin*(*sp*)_ = *hν* − [*E*_*B*_ + *Φ*_*sp*_]. (10)

*Φ_sp_* is a spectrometer constant which is calibrated using gold or copper reference [27]. Figure 5b shows the energy level diagram for the photoemission process into a vacuum or into the spectrometer. For the sample to create an electric contact with the spectrometer, the sample should be conductive and properly grounded.

Every element has a set of characteristic peaks in the XPS spectrum, which are positioned at specific binding energies. They originate from different core levels of atoms that emitted photoelectrons and have different shapes and intensities. Therefore, an analysis of the XPS spectrum allows identification of the chemical composition of the surface of the sample. The comparison of the XPS peak intensities reveals different relative concentrations of the elements on the sample surface. From the analysis of peak positions, chemical states of elements can be determined [84]. This allows for studying the electronic structure of solids and chemical reactions on the surface (for example, oxidation, destruction, etching, doping and corrosion). Modern XPS spectrometers also allow for mapping the chemical composition of the sample across its surface, the depth profiling of the chemical composition using ion beam etching and angle-resolved studies.

While the underlying principle (photoemission of electrons) is the very same in UPS and XPS, the spectroscopic information provided by the former is in many ways complementary to the latter. Due to the lower energies, the surface sensitivity of UPS is vastly superior to that of XPS. In contrast, UPS is well suited for measurements of the valence band very close to the Fermi level or even the conduction band in *n*-doped systems. In molecular systems, these include the highest occupied molecular orbitals. UPS allows characterization of adsorbed monolayers at surfaces. It can also map the electronic structure and band edges around semiconductor junctions and yields information about dopant concentration and barrier heights. On ordered surfaces of solids, angular-resolved UPS can be applied to elucidate spatial structures of extended electron states. The polarization of the incident ultraviolet light can be used to study the orientation of adsorbed molecules relative to the surface [94].

UPS is very useful technique to determine the work function of solids. As the work function is the required energy to lift an electron from the Fermi level to the vacuum state, it can be measured by the cutoff of kinetic energies in the UPS spectrum [95]. If the internal work function of the spectrometer has been calibrated and the incident photon energy is known, the work function can be directly calculated. Work function measurements are, for instance, crucial in the development of multilayered structures where the transport properties depend crucially on the proper alignment of the conduction bands. The work function is a material specific surface property and depends on the local composition and also structuring of the surface.

The C 1s XPS spectra of SWCNTs filled with MnCl_2_, MnBr_2_ [35,36], FeCl_2_, FeBr_2_, FeI_2_ [12], CoBr_2_ [13], NiCl_2_, NiBr_2_ [37], ZnCl_2_, ZnBr_2_, ZnI_2_ [14], AgCl [39], AgCl, AgBr, AgI [15], PbCl_2_, PbBr_2_, PbI_2_ [43], CdCl_2_, CdBr_2_, CdI_2_ [16], ZnCl_2_, CdCl_2_, TbCl_3_ [20], CuCl, CuBr, CuI [18], RbI [45], RbAg_4_I_5_ [46], TmCl_3_ [24], PrCl_3_ [19], HgCl_2_ [50], GaSe, GaTe [21,22], SnS, SnTe [22,23], Bi_2_Se_3_ [22], Bi_2_Te_3_ [24], Ag [24,51,53], Cu [53,54], ferrocene [56] and nickelocene [61,62] were reported.

A survey XPS spectrum of SWCNTs includes a characteristic peak of carbon at a binding energy of ~284.5 eV. A detailed C 1s XPS spectrum of SWCNTs shows a single peak, whose position and shape slightly differ for metallic and semiconducting SWCNTs. Figure 1c demonstrates the C 1s XPS spectra of 1.4 nm-diameter metallicity-sorted SWCNTs [25]. The peaks of the metallic and semiconducting SWCNTs are positioned at 284.51 and 284.57 eV binding energies. A slight difference in the peak positions of 0.06 eV for the metallicity selected SWCNTs is mainly due to differences in the chemical potentials [26,96,97]. The peak of the metallic SWCNTs has an asymmetric Doniach–Sunjic profile [98] and is slightly narrower than the peak of semiconducting SWCNTs with a symmetric Voigtian shape.

The UPS spectrum of SWCNTs is reminiscent of generic sp^2^ carbon. Figure 1d shows the valence band (VB) spectra of 1.4 nm-diameter metallicity-sorted SWCNTs [25]. The two observed dominant features in both spectra are the π-peak at 3.1 eV and σ-peak at 8.0 eV binding energy. These peaks originate from photoelectron emission from the π- and σ-bands of SWCNTs [26,99,100].

Near the Fermi level of SWCNTs, the peaks of individual van Hove singularities are observed. The spectrum of semiconducting SWCNTs shows two peaks S_1_ and S_2_ at 0.54 and 0.86 eV binding energies, respectively, which are assigned to the first and the second vHs. The spectrum of metallic SWCNTs demonstrates the peak M_1_ at 1.08 eV, which originates from the first vHs. This spectrum also reveals a significant density of states at the Fermi level.

The secondary electrons (SE) cutoff spectrum of SWCNTs features a sharp peak positioned at kinetic energies between 4.5 and 5.0 eV. The half maximum of this peak at 4.8 eV defines the work function of SWCNTs (Figure 1e) [14].

#### 2.3.2. Signatures of Doping-Induced Modifications of the PES Spectra of SWCNTs

**Figure 6:**
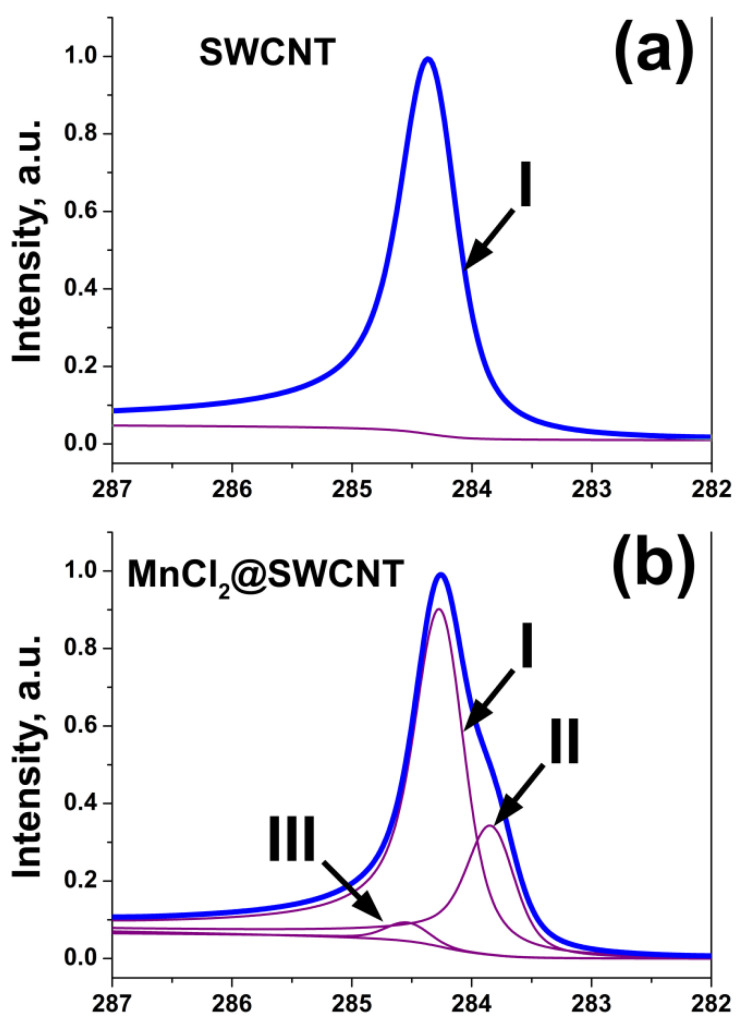
The C 1s XPS spectra of the pristine SWCNTs (**a**) and nanotubes filled with manganese chloride (**b**). The components I, II and III correspond to the unfilled, filled SWCNTs and local interactions, respectively. Reprinted by permission from [36]: SpringerNature, Applied Physics A, Kharlamova et al. Electronic properties of single-walled carbon nanotubes filled with manganese halogenides, copyright 2016.

Upon filling-induced doping of SWCNTs, their C 1s XPS spectrum can undergo the shift, broadening and increase in asymmetry of the C 1s peak. Also, the appearance of new components shifted relative to the pristine C 1s peak can be observed (Figure 6 [36], Table 3). The shift of the C 1s peak as well as new components are assigned to the alteration of the work function of SWCNTs as a result of the change of their Fermi level position due to the charge transfer between the encapsulated substances and nanotubes. The shift of the C 1s peak towards lower binding energies is caused by an increase of the work function of SWCNTs due to the lowering of their Fermi level as a result of the charge transfer from the SWCNTs to the filler, i.e., *p*-doping of SWCNTs [10]. The shift of the C 1s peak towards higher binding energies is related to a decrease of the work function of nanotubes due to an upshift of their Fermi level as a result of the charge transfer from the filler to the nanotubes, i.e., *n*-doping of SWCNTs [10]. The increase of the width and asymmetry of the C 1s peak can also be related to changes in chemical environment of filled SWCNTs [10].

**Figure 7:**
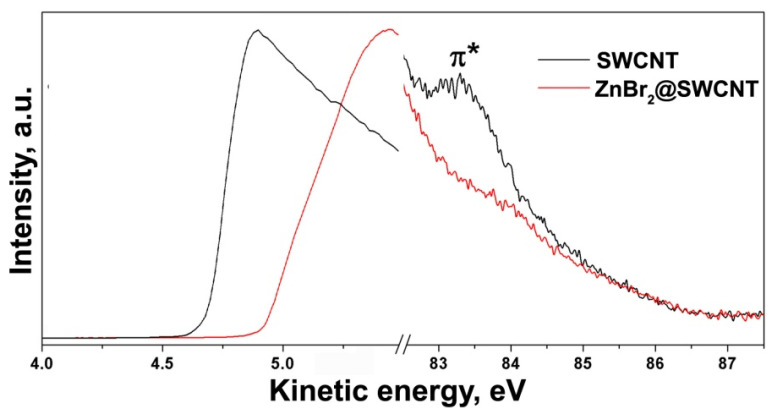
The valence band and secondary electrons cutoff spectra of the pristine SWCNTs and nanotubes filled with zinc bromide. Reprinted by permission from [14]: SpringerNature, European Physical Journal B, Kharlamova et al. Acceptor doping of single-walled carbon nanotubes by encapsulation of zinc halogenides, copyright 2012.

The valence band spectrum of filled SWCNTs reveals the shifts of the π-peak and peaks of individual vHs and their broadening as well as an increase of density of states on the Fermi level (Figure 7) [14]. The shifts of the VB peaks are a direct evidence of the change of the Fermi level position of filled SWCNTs. The shifts of these peaks towards lower or higher binding energies correspond to the decrease or increase of the Fermi level of filled SWCNTs, i.e., *p*- or *n*-doping of the nanotubes, respectively [10]. The broadening of these peaks is caused by changes in chemical environment of filled SWCNTs.

The secondary electrons cutoff spectrum of filled SWCNTs can demonstrate the shift of the main peak towards lower or higher kinetic energies (Figure 7) [14], which is attributed to the decrease or increase of the work function of filled SWCNTs due to the up- or downshift of their Fermi level, i.e., *n*- or *p*-doping of SWCNTs [10].

#### 2.3.3. Further Developments of XPS Spectroscopy of Carbon Nanotubes

Valuable information provided by photoemission spectroscopy for the investigation of carbon nanotubes made this method very popular. The quantitative data about the binding states and chemical composition of a surface of the sample are obtained for further implementation in applications. The further improvement of functionality of spectrometers and its components are needed to increase the accuracy and resolution of the X-ray photoelectron and ultraviolet photoelectron spectroscopy. The spectroscopy imaging should be developed to obtain the vast information about the sample.

### 2.4. X-ray Absorption Spectroscopy

#### 2.4.1. Basic Principle and Main Features of the XAS Spectra of SWCNTs

**Figure 8:**
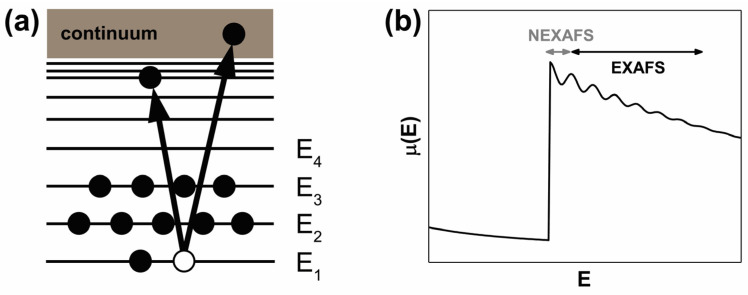
(**a**) Schematic of the transition of core electrons into an unoccupied bound state or continuum state, and (**b**) absorption coefficient *μ*(*E*) versus photon energy *E*. The spectral ranges of NEXAFS and EXAFS are labeled.

X-ray absorption spectroscopy (XAS) is used to investigate the local geometric and electronic structure of samples. The X-ray source of the choice is the very intense tunable and polarized beam available at synchrotron facilities [101]. XAS probes the energy-dependent absorption cross section of a sample in the gas, liquid or solid phase stemming from electronic core shell excitation processes. At the incident photon energy close to an atomic absorption edge, electronic transitions into unoccupied energy levels (Figure 8a) lead to distinct peaks in the XAS spectra [102]. The series of atomic adsorption edges is element specific and the K-, L- and M-edges correspond to the principal quantum numbers 1, 2 and 3, respectively.

Inside the sample, the X-ray beam is attenuated according to Beer’s law

*I_t_* = *I*_0_*e*^−*μ*(*E*)*t*^, (11)

where *I*_0_ is the incident X-ray intensity at the surface; it is the transmitted X-ray intensity, *t* is the sample thickness and *μ*(*E*) is the energy-dependent absorption coefficient [101]. In an XAS measurement the photon energy is tuned and the changes in the absorption coefficient are recorded as fine structures in the spectrum [103]. Typically, a crystalline monochromator is tuned to an energy just below an atomic absorption edge and then the photon energy is swept across and beyond the absorption edge. Once the energy is sufficient for a transition of a core electron above the Fermi level, there is a steep step in the absorption coefficient. Within several eV there are further fine structures on top of and beyond the atomic absorption edge whenever the photon energy matches a transition into an unoccupied electronic level or an unoccupied density of states in the conduction band. The actual spectra are further determined by core excitons and also the Fermi level [104].

XAS is divided into near-edge X-ray absorption fine structure (NEXAFS) that covers energies up to several tens of eV above the absorption edge and extended X-ray absorption fine structure (EXAFS) that extends from ~40 to ~800 eV above the absorption threshold (Figure 8b) [104]. NEXAFS covers resonant transitions of core electrons into unoccupied bound states (Figure 8a). Due to the relatively low kinetic energy, the excited electron is strongly scattered by neighbour atoms and its wavefunction covers the local cluster formed by the first coordination shell and eventually the second and third shells. Thus, NEXAFS probes the local site symmetry and electronic structure. The NEXAFS spectra can be used to extract information on site symmetry and chemical bonding of unknown compounds and molecular complexes [104]. For instance, the technique allows distinguishing between different crystal phases. It is also useful to investigate the local configuration of adsorbates or surface states. The method can easily distinguish the oxidation states of the absorbing atoms [103].

In the energy range of EXAFS, the core electron is no longer excited into an unoccupied bound state, but into a free or continuum state (Figure 8a). The kinetic energy of the excited core electron from the central atom is high enough that the plane-wave approximation is good and it is weakly scattered by a single neighbour atom [101]. The absorption coefficient is modulated by interference effects between the outgoing and backscattered wavefunction of the excited core electrons [104]. The details in the EXAFS oscillations are further affected by multiple scattering paths and many-body effects [101]. EXAFS measurements are used to measure interatomic distances and the coordination numbers [104].

The first XAS spectrum was reported by M. de Broglie in 1913 [105]. The fine structure beyond the atomic absorption edge was for the first time measured in 1920 by H. Fricke [106] and by G. Hertz [107]. The effects of the chemical and physical state on the fine structure in the XAS spectra was discovered by J.D. Hanawalt in 1931 [108]. The first theoretical attempt to explain the XAS fine structures was put forward in 1931 and 1932 by R.d.L. Kronig [109,110,111], and implemented successively by H. Petersen [112,113,114] and other authors in the 1930–1960s [115,116,117,118,119]. At that stage, only qualitative information could be extracted from the data. A milestone in the instrumental development in the 1960s was an improvement of commercially available diffractometers, which allowed recording much better resolved and accurately calibrated XAS spectra with conventional X-ray tubes as a source [120,121,122]. In 1971, D.E. Sayers and coauthors made the crucial advance in the interpretation of the post-edge oscillations (now referred to as EXAFS) [123]. The initial model was in the following further refined and developed by E.A. Stern, D.E. Sayers and F.W. Lytle and coauthors [124,125,126,127,128] and others [129,130,131,132,133].

Broadband synchrotron sources became available for EXAFS and NEXAFS in the 1970s, and XAS quickly proved as a reliable tool to investigate the structural and electronic configuration of molecular systems or solids. During the 1980s and 1990s, the development of software made the technique available as a useable tool to a broader scientific community for the purposes of structural characterization of materials [134].

There are three different established ways to collect the signal in an XAS measurement, namely transmission, fluorescence and electron yield modes. Transmission mode is the most direct measurement of the adsorption coefficient. The comparison of the incident and directly transmitted X-ray intensity is quantitative but requires very thin samples (e.g., gases). In contrast, fluorescence mode measures the emitted X-rays from the elements. As the incident and the probing X-rays have to traverse the material, this mode is operated on bulk samples. In partial electron yield mode, emitted electrons (either direct photoelectrons or Auger electrons) that leave the sample and overcome a voltage barrier are recorded. In total electron yield mode, the sample needs to be grounded and the drain current that replenishes any electron leaving the sample is measured. Because of the relative short mean free path of photoelectrons, electron yield mode is surface sensitive, while the two other modes are bulk sensitive [101,103].

XAS is a well-established method that is routinely used in chemistry, physics, biology, material science, engineering and earth science. The method was shown to be especially useful for the characterization of a wide range of novel materials, such as semiconductors, polymers, metalloproteins, organometallic compounds, organic materials and low dimensional nanostructures (for instance, metal nanoparticles, nanostructured metal oxides and perovskites, and carbon nanomaterials) [101,135].

In the literature, the C 1s NEXAFS spectra of SWCNTs filled with FeCl_2_, FeBr_2_, FeI_2_ [12], NiCl_2_, NiBr_2_ [37], ZnCl_2_, ZnBr_2_, ZnI_2_ [14], CdCl_2_, CdBr_2_, CdI_2_ [16], AgCl, AgBr, AgI [15], CuCl, CuBr, CuI [18], HgCl_2_ [50] were reported. The NEXAFS spectrum of SWCNTs at C 1s edge has an overall shape that is reminiscent for graphite [26] and features two characteristic resonances: the π*-resonance at a photon energy of ~285 eV and the σ*-resonance at a photon energy of ~292 eV. They correspond to transitions of a C 1s core electron to the unoccupied π*- and σ*-bands of nanotubes, respectively [26]. Figure 1f demonstrates the C 1s NEXAFS spectra of metallicity-mixed semiconducting and metallic SWCNTs [26]. A clear fine structure is observed in the π*- resonance, which can be related to the diameter-dependent one dimensional vHs singularities in the unoccupied density of states of the SWCNTs [96].

#### 2.4.2. Signatures of Doping-Induced Modifications of the XAS Spectra of SWCNTs

**Figure 9:**
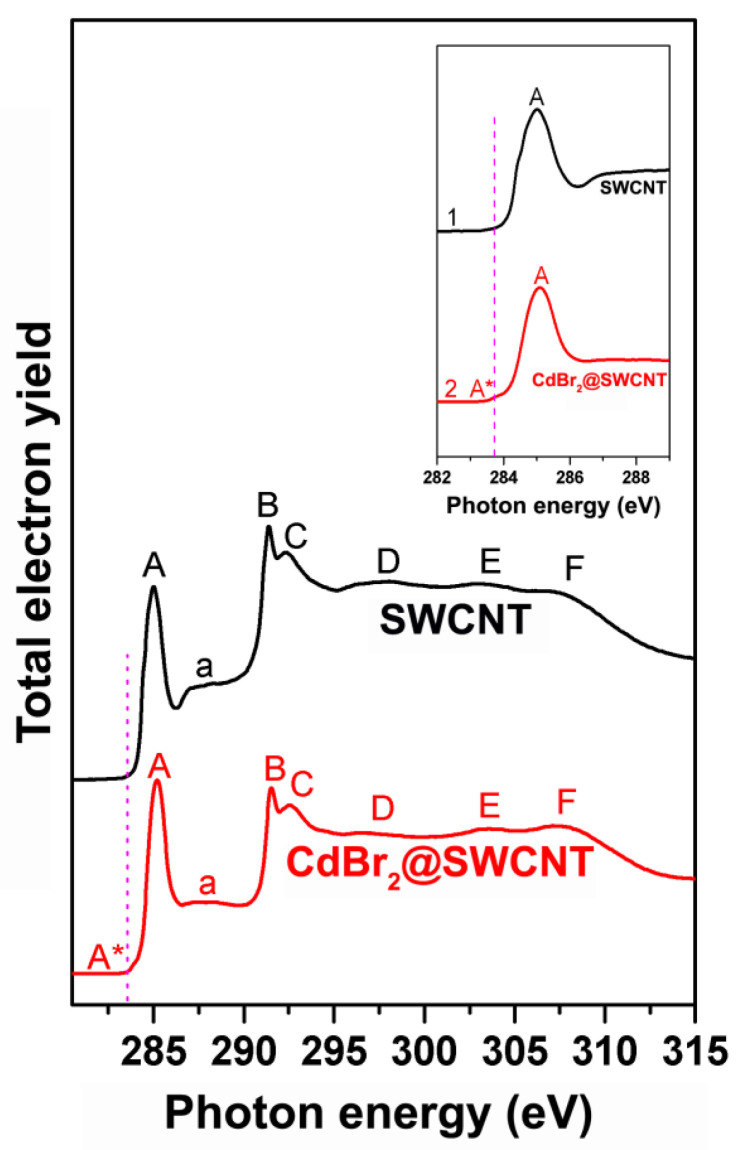
The C 1s NEXAFS spectra of the pristine SWCNTs and nanotubes filled with cadmium bromide. The inset zooms in the π*-resonance. The dashed vertical line marks the new feature A*. Reprinted by permission from [16]: SpringerNature, Journal of Materials Science, Kharlamova et al. Charge transfer in single-walled carbon nanotubes filled with cadmium halogenides, copyright 2013.

Upon filling of SWCNTs, their C 1s NEXAFS spectrum is expected to mostly retain the spectrum of the pristine nanotubes; however, the origin of an additional pre-edge peak at ~1 eV lower photon energies than the π*-resonance can be observed (Figure 9 [16], Table 4). This new peak is due to transitions of a C 1s core electron to new localized states that are formed as a result of the hybridization of the π-orbitals of atoms of SWCNTs with orbitals of atoms of the incorporated substances, i.e., the formation of chemical bonds between nanotubes and filler [10].

#### 2.4.3. Further Developments of the XAS Spectroscopy of Carbon Nanotubes

The investigation of the electronic structure of carbon nanotubes and filled carbon nanotubes by X-ray absorption spectroscopy gives the information on bonding environment and chemical state. The next step would be the obtaining of the polarization dependence of XAS spectra for the increased informativeness. The theoretical modelling of the XAS spectra will be other further direction.

## 3. Conclusions

In this review, the spectroscopic techniques for the analysis of the electronic properties of filled SWCNTs are discussed. Optical absorption spectroscopy, Raman spectroscopy, photoemission spectroscopy and X-ray absorption spectroscopy are considered. For these techniques, the basic principle and main features of SWCNTs as well as signatures of doping-induced modifications of filled SWCNT spectra are presented. This review will stimulate further development of methods for investigating filled SWCNTs to implement their full potential in applications.

## 4. Outlook

The synthesis and separation of metallic and semiconducting or even single chiralities of SWCNTs vastly extends the scope of possible applications for filled SWCNT. This is true especially for applications that rely on specific electronic properties. For this, the further development of spectroscopic methods of investigation of the electronic properties is required. If there is more control on electronic properties of the starting material, then there is also more control on the achievable results if the electronic properties are modified by filling SWCNTs. This new level of attainable accuracy in engineering electronic properties by choosing a type or even single chirality of SWCNTs as well as an appropriate dopant for filling will greatly benefit most of the applications.

There are doubtlessly a number of open challenges for applications of filled SWCNTs. Considering the recent advancements in purification, synthesis and filling of SWCNTs, however, many of these challenges can be expected to be overcome in the near future. Once this is no longer an issue, there will be most likely a plethora of new ideas for more potential applications.

## Figures and Tables

**Table 1:** Summary of doping-induced modifications of the OAS spectra of filled SWCNTs.

Filled Substance	Observed Modification of the OAS Spectrum	Reference
FeCl_2_, FeBr_2_, FeI_2_	$\mathrm{Suppression}\;\mathrm{of}\;\mathrm{the}\;E_{11}^{S}$ peak	[12]
CoBr_2_	$\mathrm{Suppression}\;\mathrm{of}\;\mathrm{the}\;E_{11}^{S}$ peak	[13]
ZnCl_2_, ZnBr_2_, ZnI_2_	$\mathrm{Suppression}\;\mathrm{of}\;\mathrm{the}\;E_{11}^{S}$ peak	[14]
AgCl, AgBr, AgI	$\mathrm{Suppression}\;\mathrm{of}\;\mathrm{the}\;E_{11}^{S}$ peak	[15]
CdCl_2_, CdBr_2_, CdI_2_	$\mathrm{Suppression}\;\mathrm{of}\;\mathrm{the}\;E_{11}^{S}$ peak	[16]
CuCl	$\mathrm{Suppression}\;\mathrm{of}\;\mathrm{the}\;E_{11}^{S}$ peak	[17]
CuCl, CuBr, CuI	$\mathrm{Suppression}\;\mathrm{of}\;\mathrm{the}\;E_{11}^{S}$ peak	[18]
PrCl_3_	$\mathrm{Suppression}\;\mathrm{of}\;\mathrm{the}\;E_{11}^{S}$ peak	[19]
TbCl_3_	$\mathrm{Suppression}\;\mathrm{of}\;\mathrm{the}\;E_{11}^{S}$ peak	[20]
GaSe, GaTe	$\mathrm{Suppression}\;\mathrm{of}\;\mathrm{the}\;E_{11}^{S}$ peak	[21,22]
SnS, SnTe	No modifications	[22,23]
Bi_2_Se_3_	No modifications	[22]
Bi_2_Te_3_	No modifications	[24]

**Table 2:** Summary of doping-induced modifications of the Raman spectra of filled SWCNTs.

Filled Substance	Observed Modification of the Raman Spectrum	Reference
MnCl_2_, MnBr_2_	Shifts and changes in relative intensities of peaks in the RBM and G-bands	[35,36]
FeCl_2_, FeBr_2_, FeI_2_	Shifts and changes in relative intensities of peaks in the RBM and G-bands	[12]
CoBr_2_	Shifts and changes in relative intensities of peaks in the RBM and G-bands	[13]
NiCl_2_, NiBr_2_	Shifts and changes in relative intensities of peaks in the RBM and G-bands	[37]
ZnCl_2_	Shifts and changes in relative intensities of peaks in the RBM and G-bands	[20]
ZnCl_2_, ZnBr_2_, ZnI_2_	Shifts and changes in relative intensities of peaks in the RBM and G-bands	[14]
AgCl, AgBr, AgI	Shifts and changes in relative intensities of peaks in the RBM and G-bands	[15]
AgCl	Shifts and changes in relative intensities of peaks in the RBM and G-bands	[38,39]
CuCl	Shifts and changes in relative intensities of peaks in the RBM and G-bands	[17]
CuI	Shifts and changes in relative intensities of peaks in the RBM and G-bands	[40,41]
CuCl, CuBr, CuI	Shifts and changes in relative intensities of peaks in the RBM and G-bands	[18]
CdCl_2_	Shifts and changes in relative intensities of peaks in the RBM and G-bands	[20,42]
CdCl_2_, CdBr_2_, CdI_2_	Shifts and changes in relative intensities of peaks in the RBM and G-bands	[16]
PbCl_2_, PbBr_2_, PbI_2_	Shifts and changes in relative intensities of peaks in the RBM and G-bands	[43]
SnF_2_	No modifications	[44]
RbI	Shifts and changes in relative intensities of peaks in the RBM and G-bands	[45]
RbAg_4_I_5_	Shifts and changes in relative intensities of peaks in the RBM and G-bands	[46]
TbCl_3_	Shifts and changes in relative intensities of peaks in the RBM and G-bands	[20,47,48]
TbBr_3_, TbI_3_	Shifts and changes in relative intensities of peaks in the RBM and G-bands	[48]
TmCl_3_	Shifts and changes in relative intensities of peaks in the RBM and G-bands	[24,47]
PrCl_3_	Shifts and changes in relative intensities of peaks in the RBM and G-bands	[19,47]
LuCl_3_, LuBr_3_, LuI_3_	Shifts and changes in relative intensities of peaks in the RBM and G-bands	[49]
HgCl_2_	Shifts and changes in relative intensities of peaks in the RBM and G-bands	[50]
GaSe, GaTe	Shifts and changes in relative intensities of peaks in the RBM and G-bands	[21,22]
SnS, SnTe	No modifications	[22,23]
Bi_2_Se_3_	Slight modifications	[22]
Bi_2_Te_3_	Slight modifications	[24]
Ag	Shifts and changes in relative intensities of peaks in the RBM and G-bands	[24,51,52,53]
Cu	Shifts and changes in relative intensities of peaks in the RBM and G-bands	[53,54]
Ferrocene	No modifications	[55,56,57,58]
Cobaltocene	No modifications	[59,60]
Nickelocene	No modifications	[61,62,63]

**Table 3:** Summary of doping-induced modifications of the C 1s XPS spectra of filled SWCNTs.

Filled Substance	Observed Modification of the C 1s XPS Spectrum	Reference
MnCl_2_, MnBr_2_	Appearance of new downshifted components and broadening	[35,36]
FeCl_2_, FeBr_2_, FeI_2_	Appearance of new downshifted components and broadening	[12]
CoBr_2_	Appearance of new downshifted components and broadening	[13]
NiCl_2_, NiBr_2_	Appearance of new downshifted components and broadening	[37]
ZnCl_2_, ZnBr_2_, ZnI_2_	Appearance of new downshifted components and broadening	[14]
AgCl	Downshift, broadening and increase in asymmetry	[39]
AgCl, AgBr, AgI	Appearance of new downshifted components and broadening	[15]
PbCl_2_, PbBr_2_, PbI_2_	Downshift, broadening and increase in asymmetry	[43]
CdCl_2_, CdBr_2_, CdI_2_	Appearance of new downshifted components and broadening	[16]
ZnCl_2_, CdCl_2_, TbCl_3_	Appearance of new downshifted components and broadening	[20]
CuCl, CuBr, CuI	Appearance of new downshifted components and broadening	[18]
RbI	Upshift, broadening and increase in asymmetry	[45]
RbAg_4_I_5_	Appearance of new downshifted components and broadening	[46]
TmCl_3_	Appearance of new downshifted components and broadening	[24]
PrCl_3_	Appearance of new downshifted components and broadening	[19]
HgCl_2_	Downshift, broadening and increase in asymmetry	[50]
GaSe, GaTe	Appearance of new downshifted components and broadening	[21,22]
SnS, SnTe	Broadening	[22,23]
Bi_2_Se_3_	Broadening	[22]
Bi_2_Te_3_	Broadening	[24]
Ag	Appearance of new upshifted components and broadening	[24,51,53]
Cu	Appearance of new upshifted components and broadening	[53,54]
Ferrocene	Upshift, broadening and increase in asymmetry	[56]
Nickelocene	Uphift, broadening and increase in asymmetry	[61,62]

**Table 4:** The summary of doping-induced modifications of the C 1s NEXAFS spectra of filled SWCNTs.

Filled Substance	Observed Modification of the C 1s NEXAFS Spectrum	Reference
FeCl_2_, FeBr_2_, FeI_2_	Appearance of additional component below π-resonance	[12]
NiCl_2_, NiBr_2_	Appearance of additional component below π-resonance	[37]
ZnCl_2_, ZnBr_2_, ZnI_2_	Appearance of additional component below π-resonance	[14]
CdCl_2_, CdBr_2_, CdI_2_	Appearance of additional component below π-resonance	[16]
AgCl, AgBr, AgI	Appearance of additional component below π-resonance	[15]
CuCl, CuBr, CuI	Appearance of additional component below π-resonance	[18]
HgCl_2_	Appearance of additional component below π-resonance	[50]

## Data Availability

The data are available on request from the author.
